# Editorial: Extracellular Vesicles in Bone Oncology

**DOI:** 10.3389/fonc.2022.861335

**Published:** 2022-03-03

**Authors:** Francesca Perut, Roberta Tasso, Bettina Mannerström

**Affiliations:** ^1^ Biomedical Sciences and Technologies (BST), Biomedical Sciences and Technologies Lab, IRCCS Istituto Ortopedico Rizzoli, Bologna, Italy; ^2^ Department of Experimental Medicine (DIMES), University of Genova, Genova, Italy; ^3^ Department of Oral and Maxillofacial Diseases, University of Helsinki and Helsinki, University Hospital, Helsinki, Finland

**Keywords:** extracellular vesicles, bone tumors, drug delivery, liquid biopsy, tumor microenvironment, metastasis

Extracellular vesicles (EVs), which consist of a group of heterogeneous nanosized vesicles secreted by almost all types of cells, have received significant interest in the past few years. Their crucial role in cell-to-cell communication has been explored in different types of tumors (1), and EV cargo has been associated with cancer growth, metastasis, and chemoresistance (2).

The activity of the EV in the bone tumor microenvironment is not limited to the relationship between cells (osteoblasts, osteoclasts, endothelial cells, cancer stem cells, fibroblasts/stromal cells, immune cells, etc.), but is affected and influenced itself by the complexity of the bone microenvironment signals (oxygen tension, mechanical loading, etc.).

This Research Topic of Frontiers in Oncology offers updated perspectives on the specific roles of EVs in bone oncology, including both primary bone tumors and bone metastasis.

The role of EVs in pre-metastatic niche formation and organotropic metastasis has recently been extensively described by Rezaie et al. (3). The authors highlighted the role of EVs in the interactions between tumor cells and the metastatic microenvironment and in the reawakening of dormant niches. In skeletal metastases, the homeostatic bone remodeling process is unbalanced, leading to an osteolytic and osteoblastic metastatic phenotype depending on the differentiation and activation of osteoblasts, pre-osteoblasts, and osteoclasts.

Li and Wang summarize recent literature describing the activity of tumor-derived EVs in bone metastases and their potential molecular mechanisms in prostatic, breast, and lung cancer, as well as in multiple myeloma and acute myelocytic leukemia. EVs contain pro-osteoclastogenic molecules that induce osteoclastogenesis and osteolytic metastasis and transport various osteoblast-stimulating factors that mediate osteoblast differentiation and osteoblastic metastasis in the bone.

The key role of EVs in the osteoblast-osteoclast crosstalk has been ascertained in the bone metastatic microenvironment. The role of EV in bone remodeling is also assessed in a physiological context in reviews by Cappariello and Rucci and by Urciuoli and Peruzzi. Here, the authors describe how this physiological “virtuous cycle” is converted into a “vicious cycle” in the tumoral context where cancer cells exploit the EV information transfer and molecular pathway activation to their advantage. Moreover, the authors point out how bone sarcoma cells use EVs to boost growth in the bone and how they educate the bone microenvironment to host metastases.

In the Ewing sarcoma context, Pachva et al. described in detail how tumor EVs may mediate the reprogramming of fibroblasts into cancer-associated fibroblasts and how they may prompt pro-inflammatory cytokine production by tumor-associated macrophages and dendritic cells and inhibit T cell-mediated response associated with cancer progression.

It is worth noting that various species of non-coding RNA, including long non-coding RNA (lncRNA), are contained in the EV cargo (4). The subcellular locations of lncRNAs determine the function they can perform, but the role of lncRNAs carried by EVs is still under debate. Xiong et al. show that the lncRNA *FOXP4*-*AS1* is an unfavorable prognostic factor in Ewing sarcoma and may modulate the tumor immune microenvironment in an EV-mediated manner.

Among the several different microRNAs (miRNAs) that were identified in bone tumor-derived EVs, Araki et al. highlight the presence of miR-146a-5p in osteosarcoma derived EV cargo and describe the mechanisms by which EVs from highly malignant osteosarcoma cells reduce osteoclast maturation.

EVs are natural nanocarriers, and several groups are working with considerable efforts on loading chemotherapeutic drugs into EVs to target bone tumor cells and simultaneously decrease drug toxicity (5). Yang et al. showed some interesting preliminary studies in the osteosarcoma context.

Emerging evidence suggests that circulating EVs also represent an intriguing biomarker with potentially significant applications in cancer diagnostics and importantly, for monitoring patients with recurrent disease. Identification of the specific markers in the EV cargo is achieving significant results (6, 7), yet, the applicability is hampered by the lack of methodology for easy and reliable EV isolation. To this end, Xu et al. presented a ZnO-nanorod integrated microfluidic chip for the quantification of osteosarcoma plasma derived-EVs associated with vimentin expression.

Mechanical stimulation and loading are specific features of bone tissue remodeling and, as suggested by Urciuoli and Peruzzi, the mechanoenvironment as an additional component in the relationship between EVs and cells in the bone requires appropriate consideration. Mechanical stimulation may influence EV release and regulate the cargo of the EVs. Moreover, EV signaling by means of induction of matrix metalloproteinases expression or by the transport of lytic enzyme cargo may actively participate in extracellular matrix remodeling during cancer progression.

Taken together, the original research and review contributions of this Research Topic provide an overview of the emerging and ongoing research progress on the role of EVs in bone oncology, by considering the interaction between cells, the mechanical loading of the bone microenvironment, and future applications in terms of diagnostic/prognostic biomarkers and drug delivery.

## Author Contributions

All authors contributed equally to the writing of the Editorial manuscript.

## Conflict of Interest

The authors declare that the research was conducted in the absence of any commercial or financial relationships that could be construed as a potential conflict of interest.

## Publisher’s Note

All claims expressed in this article are solely those of the authors and do not necessarily represent those of their affiliated organizations, or those of the publisher, the editors and the reviewers. Any product that may be evaluated in this article, or claim that may be made by its manufacturer, is not guaranteed or endorsed by the publisher.
